# Cerebral fat embolism treated with mechanical embolectomy: case report and systematic review of the literature

**DOI:** 10.1093/bjrcr/uaag019

**Published:** 2026-05-06

**Authors:** Rohan Mudkavi, Tatyana Sarnecki, Ei Ei Phyu, Htet Phyo Than, Veronica Phillips, Naeem Ahmad, Smriti Agarwal, Yogish Joshi, Dilina Rajapakse, Abhishekh H Ashok

**Affiliations:** The School of Clinical Medicine, University of Cambridge, Cambridge CB2 0SP, United Kingdom; Department of Radiology, Cambridge University Hospitals NHS Foundation Trust, Cambridge CB2 0QQ, United Kingdom; Department of Geriatric Medicine, Cambridge University Hospitals NHS Foundation Trust, Cambridge CB2 0QQ, United Kingdom; Department of Geriatric Medicine, Cambridge University Hospitals NHS Foundation Trust, Cambridge CB2 0QQ, United Kingdom; University of Cambridge Medical Library, University of Cambridge, Cambridge CB2 0SP, United Kingdom; Department of Stroke Medicine, Cambridge University Hospitals NHS Foundation Trust, Cambridge CB2 0QQ, United Kingdom; Department of Stroke Medicine, Cambridge University Hospitals NHS Foundation Trust, Cambridge CB2 0QQ, United Kingdom; Department of Clinical Neurosciences, University of Cambridge, Cambridge CB2 0QQ, United Kingdom; Department of Radiology, Cambridge University Hospitals NHS Foundation Trust, Cambridge CB2 0QQ, United Kingdom; Department of Radiology, Cambridge University Hospitals NHS Foundation Trust, Cambridge CB2 0QQ, United Kingdom; Department of Radiology, Cambridge University Hospitals NHS Foundation Trust, Cambridge CB2 0QQ, United Kingdom; Department of Radiology, University of Cambridge, Cambridge CB2 0QQ, United Kingdom

**Keywords:** cerebral fat embolism, thrombectomy, embolectomy, aspiration, stroke

## Abstract

Cerebral fat embolism (CFE) is a rare complication of orthopaedic, cardiac and plastic surgery and major trauma. Mechanical embolectomy has been used as a treatment option for this condition. Here, we described a case report of an elderly woman who developed CFE after a total hip replacement which was treated with mechanical embolectomy with aspiration alone. The patient developed left-sided hemiparesis and abnormal posturing after surgery and a CT angiogram 45 minutes later showed a right M1 embolus with fat attenuation. Mechanical embolectomy with aspiration was conducted to produce a TICI score of 3. No patent foramen ovale (PFO) was found to explain the paradoxical embolism. Subsequently, a systematic literature review on the use of mechanical embolectomy for CFE was conducted, with the databases Medline, Web of Science, Embase, Cinahl, and Scopus being searched. The review found 20 cases, 13 of which were treated with aspiration and a stent retriever, and 5 of which were treated with aspiration only. A reduced mortality was observed in the aspiration only cohort compared to the aspiration plus stent retriever cohort, despite both groups having similar age and gender. Among patients treated with stenting, 3 out of 13 (23%) died, compared to 0 out of 5 (0%) patients treated with aspiration alone. Although this difference was not statistically significant, it may reflect a trend worth further exploration, potentially limited by the small sample size. Further registry-based studies and preclinical models are required to elucidate the effectiveness and superior technique of mechanical embolectomy for CFE.

## Introduction

Cerebral fat embolism (CFE) is a rare but potentially fatal phenomenon. The condition can occur after trauma with open fractures,^1^ or after surgery, particularly plastic surgery,^2^ and orthopaedic surgery.^3^ CFE arises when fat droplets enter the bloodstream before travelling to the brain to block a cerebral blood vessel, causing ischaemia and subsequent infarction of the vascular territory supplied by said vessel. There are 2 broad theories of how lipids enter the blood to cause fat embolism—the mechanical and biochemical theories. The mechanical theory hypothesizes that injury causes an increased intramedullary pressure, allowing fat in bone marrow to enter injured venous sinusoids—this fat subsequently travels through arteriovenous shunts (such as a patent foramen ovale) or straight through the pulmonary circulation to reach the systemic circulation. The biochemical theory states that stress from trauma or surgery causes hormonal changes, particularly increased catecholamines, which results in free fatty acids being systemically released and fat droplets being produced.^4^

Mechanical thrombectomy/embolectomy is a treatment option for acute ischaemic stroke which involves the endovascular mechanical removal of a thrombus either through aspiration alone or aspiration with a stent retriever. Although mechanical embolectomy has been discussed and implemented as a potential management option for CFE,^3^^,^^5^^,^^6^ a formal systematic literature review on this subject has not been carried out. Indeed, it is unclear which technique of mechanical embolectomy is superior for CFE. Given that a fat embolus is more friable and softer than a thrombus, assessing the superiority of aspiration or a stent retriever with aspiration would be useful in the treatment decision. Here, we firstly present the case of a woman in her 70 s who developed a CFE during a right total hip replacement, which was treated with mechanical embolectomy. This is followed by a discussion of the literature surrounding mechanical embolectomy as a treatment option for CFE, with a focus on the technique of embolectomy used.

## Case report

A right-handed lady in her 70 s with a history of atrial fibrillation, ischaemic stroke, gastro-oesophageal-reflux-disease, hypertension, and osteoarthritis presented for an elective right total hip replacement. The patient had a spinal block with 3 mL levobupivacaine, and sedation with a low dose (20 mg) propofol infusion. Towards the end of the procedure, after the surgeons had added the cement and prosthetic and were closing the operative site, the patient was noted to be moving and behaving normally for the degree of sedation she was under.

**Figure 1.**
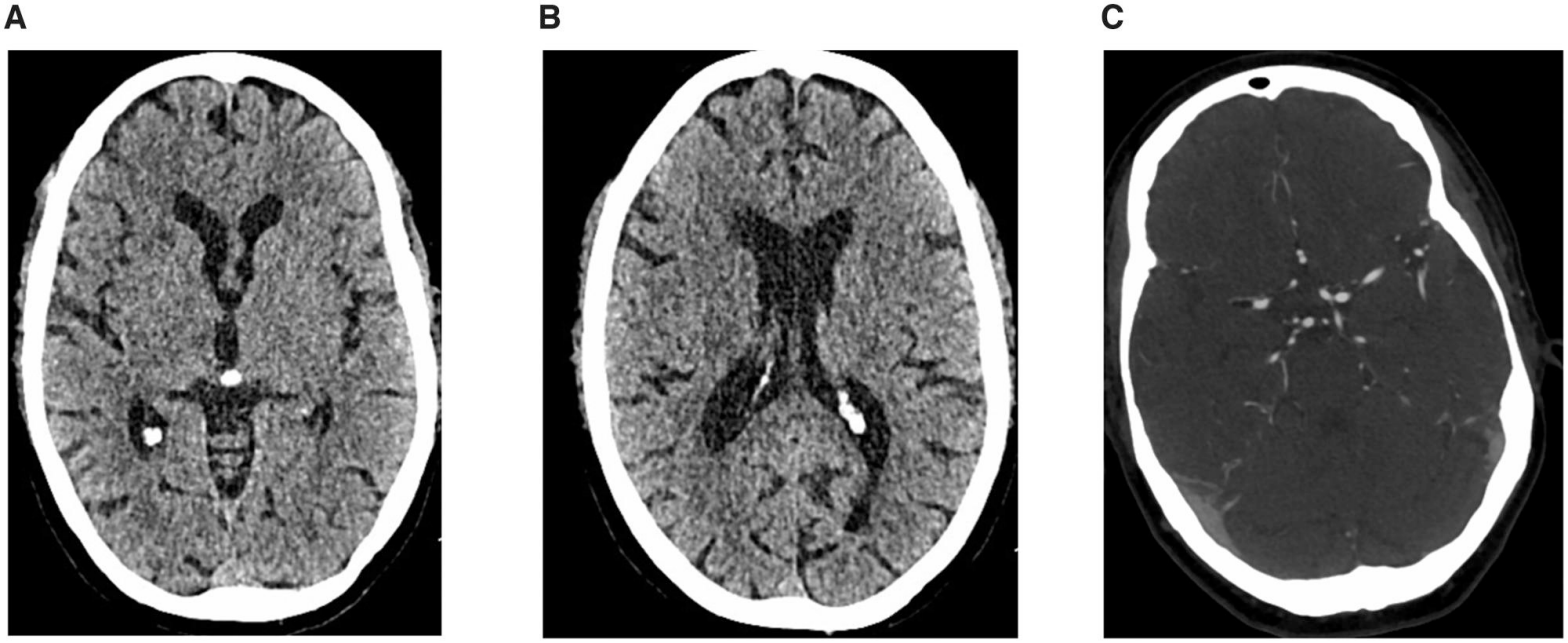
Initial imaging investigations. (A) CT head at ganglionic level showed a small focus of slightly decreased attenuation in the medial part of the right thalamus. (B) CT head at supraganglionic level revealed no obvious acute abnormalities. (C) CT angiogram showed a right M1 embolus with fat attenuation.

Shortly after this, she became hypoxic, tachypnoeic, hypertensive and tachycardic—sedation and vasopressor input were hence ceased. Following the application of a dressing and moving the patient to her bed from the theatre, she was noted to have a decreased level of consciousness despite cessation of the sedation. She also had abnormal posturing, was not moving her left side and had a GCS of 5 (E1V1M3). Hence, the patient was immediately intubated and ventilated, and a propofol/remifentanil/metaraminol infusion was started. A CT head and CT angiogram were conducted, 45 minutes after the initial symptoms were noted. The CT head showed a small focus of slightly decreased attenuation in the medial part of the right thalamus, consistent with an area of acute infarction (Figure 1A). Ganglionic and supraganglionic images revealed an ASPECTS score of 10 (Figure 1A,B). The CT angiogram showed a right M1 embolus with fat attenuation (Figure 1C) with no internal carotid, common carotid or vertebral artery stenoses. A decision was made to proceed to mechanical embolectomy.

**Figure 2.**
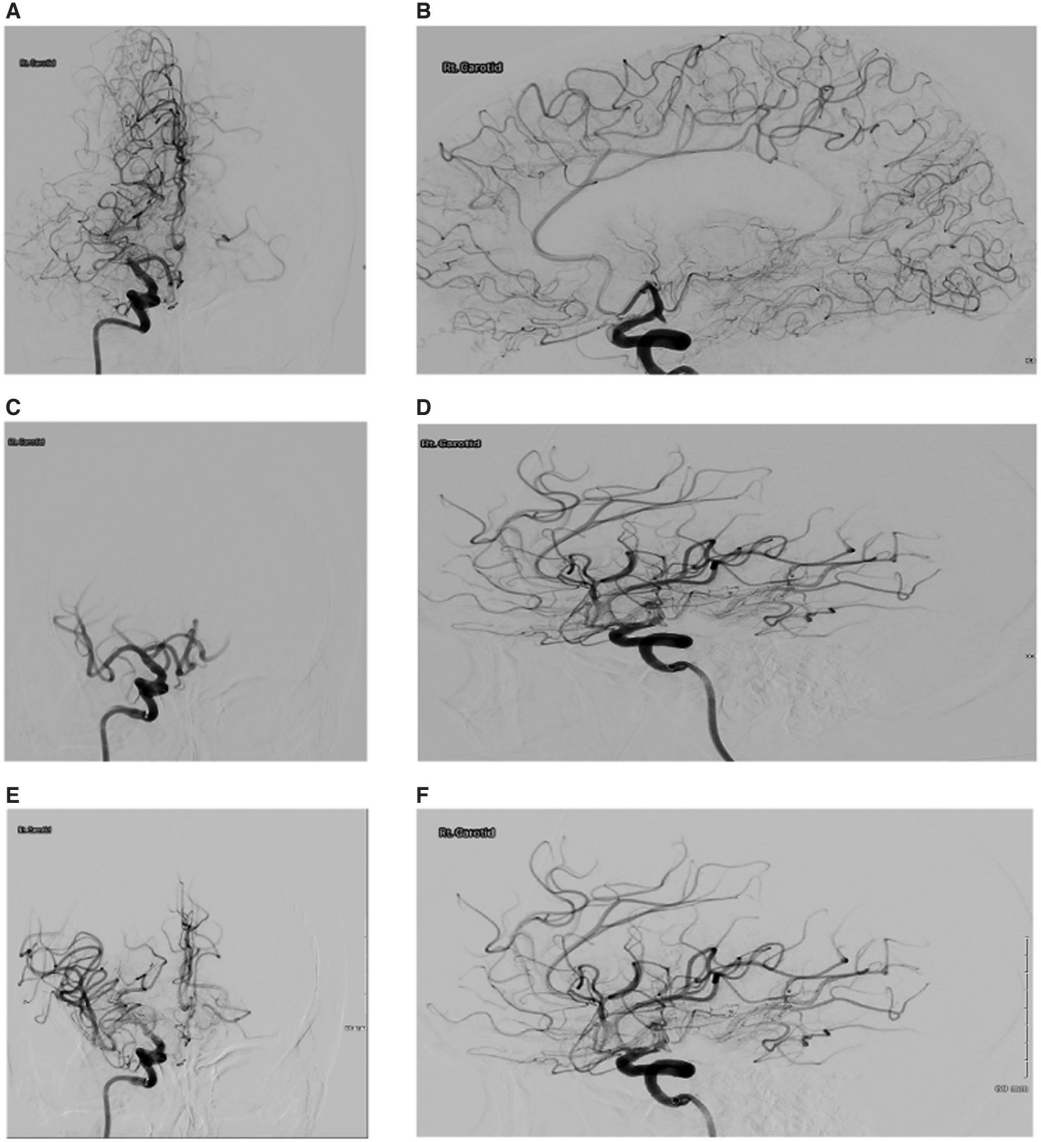
Mechanical embolectomy imaging. (A and B) Pre-aspiration, showing right M1 embolus. (C-F) Post-aspiration showing increase in blood flow through the M1 segment.

The patient arrived in the angiography suite approximately 1 hour 20 minutes after the symptoms were first noticed. The procedure was performed under general anaesthesia. Ultrasound-guided access of the right common femoral artery was obtained, and an 8F arterial sheath was inserted. A Cerebase guiding catheter (Cerenovus, Johnson & Johnson MedTech, Irvine, CA, USA), with VTK catheter over a Terumo guidewire (Terumo Corporation, Tokyo, Japan) was advanced into the right internal carotid artery. Selective angiography demonstrated an occlusion of the right M1 segment of the middle cerebral artery (Figure 2A,B). A 6F Cereglide aspiration catheter (Cerenovus, Johnson & Johnson MedTech, Irvine, CA, USA) was navigated to the face of the thrombus, and aspiration was performed using the VacLok aspiration system (Merit Medical, South Jordan, UT, USA). A macroscopic fat embolus was successfully retrieved. Final angiography showed complete reperfusion with a Thrombolysis in Cerebral Infarction (TICI) grade 3 result (Figure 2C-F). The procedure duration was approximately 20 minutes. The aspirated contents were sent to pathology, who later confirmed a fat embolus based on the histology.

**Figure 3.**
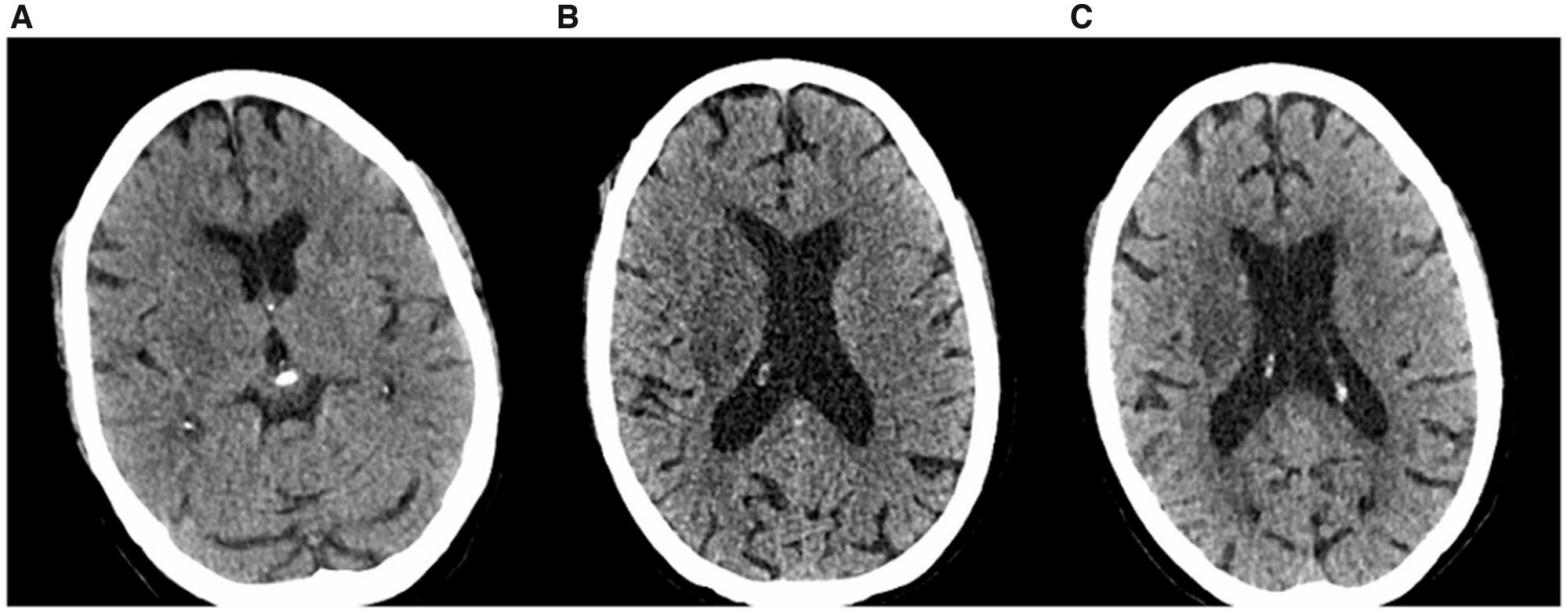
Progression of infarct. (A) CT head taken 1 day post-admission showing evolving ischaemia in lenticulostriate territory. (B) CT head taken 6 days post-admission showing development of this ischaemia. (C) CT head taken at 13 days post-admission showing further, expected evolution.

Following the mechanical embolectomy, interval imaging was obtained to assess the progression of ischaemic changes. A CT head taken 1 day post-admission showed evolving ischaemia in the right lenticulostriate territory, with the remainder of the MCA territory largely spared (Figure 3A). Repeat imaging 6 days post-admission showed development of this ischaemia laterally into the insular ribbon and inferiorly into the superior temporal lobe (Figure 3B). Further imaging at 13 days post-admission showed further expected evolution of the ischaemic changes (Figure 3C).

A transthoracic echocardiogram was conducted 2 days post-admission to investigate a possible PFO to explain the paradoxical embolism. However, the echo noted no such abnormality. An increased pulmonary arterial pressure and mild-moderate tricuspid regurgitation was noted. A bubble contrast echocardiogram has a higher sensitivity for detecting PFOs, but this was not performed.

Clinically, the patient was immediately extubated following embolectomy. However, she had a low GCS of 7 (E1 V1 M5), increased secretions, her left side was not moving asides from a flicker of movement in the left foot, and her sensorium was poor. Hence, she was re-intubated, sedated and monitored in the neurocritical care unit (NCCU) overnight. The patient’s GCS improved to 10 (E3 V1 M6) by day 2 post-admission, and extubation was successfully carried out on day 3. On day 5 review, the patient had a GCS of 11 (E3V2M6), could not communicate, was minimally alert, and had a left-sided neglect, a right-sided gaze deviation, and a dense left hemiplegia. The patient was transferred from NCCU to a stroke unit for rehabilitation with physiotherapy, occupational therapy and speech and language therapy. Over the next 2 months, the patient improved to the point where she was alert, could communicate fully, and mobilize with an electrical wheelchair. In addition, a newly developed post-stroke depression was successfully treated with fluoxetine. However, the gaze deviation, hemiplegia, and neglect largely persisted.

## Literature review

### Methods

The databases Medline (via Ovid), Embase (via Ovid), Web of Science (Core Collection), Scopus, and Cinahl (via Ebscohost) were searched from January 2015 to October 2024 by V.P. The Peer Review of Electronic Search Strategies (PRESS) checklist was utilized by 2 librarian colleagues of V.P. to peer-review the search strategy.^7^ The search strategy was evaluated with PRISMA-S guidelines.^8^ V.P. searched databases separately, rather than searching multiple databases on the same platform. For each database the search syntax was modified to account for differences in thesaurus terms and controlled vocabulary between databases. Deduplication was conducted in Endnote 21 by V.P., as per the method in Bramer et al.^9^ The search strategy used in each database is documented (see Appendix S1). Deduplicated results (258 publications, Table 1) were uploaded into Rayyaan. The review was not registered and a registered protocol was not prepared. All the data obtained from included studies are publicly available via the online databases searched.

**Table 1.** Papers found using search strategy in different databases.

Database	No. of papers
Medline	78
Embase	166
Web of Science	56
Scopus	113
Cinahl	12
After deduplication	258

**Figure 4.**
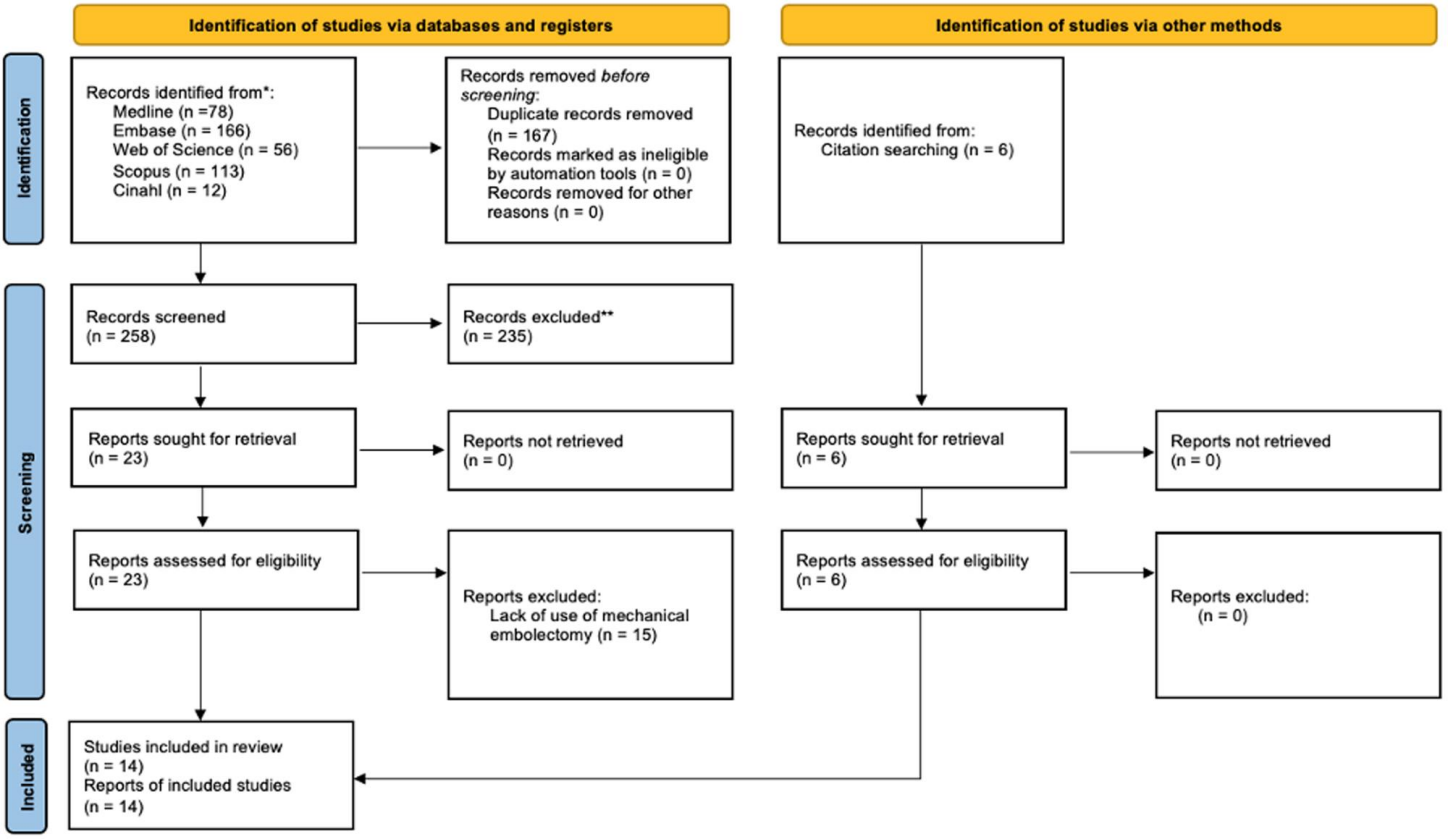
PRISMA flowchart.

Both abstract and full text screening were conducted on Rayyan by 2 colleagues independently (T.S. and R.M.) based on inclusion and exclusion criteria. Inclusion criteria were: having neurological signs of a cerebral stroke; having imaging or histological signs of cerebral large vessel occlusion caused by fat; having treatment with mechanical embolectomy. Exclusion criteria were: patients with fat embolism syndrome (ie, disseminated fat droplets); respiratory failure preventing mechanical embolectomy from occurring; non-fat emboli (ie, cholesterol, thrombi); fat embolism treated conservatively or by non-endovascular invasive measures. Any disagreements on inclusion were resolved by a third colleague (A.H.A.). We applied no language limitations. The literature review yielded 8 studies, with 6 more coming from citation searching within these studies. Overall, this yielded 14 studies with 20 cases of CFE treated with mechanical embolectomy (Figure 4). One article^10^ published in Chinese was translated using Google Translate in October 2024.

The overall cohort of cases was characterized in terms of age, gender, cause of CFE, occluded artery, and presence of a PFO. Subsequently, cases treated with aspiration alone were compared to those treated with aspiration plus a stent retriever. When comparing average age, change in NIHSS and final mRS, the normality of distribution was determined using a Shapiro-Wilk test—an unpaired *t*-test and Mann-Whitney *U* test were used to determine significance in normally and non-normally distributed data, respectively. When comparing gender proportions and mortality rates, Fisher’s exact test was used to determine significance due to small sample sizes.

## Results

### A table of CFEs in the literature

The results of the literature review are shown (Tables 2 and 3).

**Table 2.** Background of patient, cause, and location of CFE, presence/absence of a PFO, and presence/absence of synchronous systemic fat embolization for each case.

Reference number	Age	Gender	Cause of fat embolism	PFO confirmed?	Artery occluded	Synchronous systemic fat embolization?
^2^	31	F	LS and bilateral temporal and tear troughs FG		L-ICA, L-ECA	N
^3^	71	M	LKR (general anaesthesia)	Y	L-ICA, L-MCA-M1	N
^3^	47	M	Open Mechanical MVR	N	Basilar artery-mid	N
^5^	56	M	RKR (spinal anaesthesia)	Y	L-MCA-M1	N
^6^	22	F	Bilateral temporal FG		R-ICA, R-MCA-M1	N
^11^	70	M	Spontaneous		R-ICA, R-MCA	N
^12^	33	F	LS and bilateral gluteal FG	N	R-MCA	N
^12^	80	M	Spontaneous	N	Basilar artery, L-V4	N
^13^	69		Open Mechanical MVR		R-ICA, R-MCA-M1	N
^14^	70	F	Right THA	Y	Basilar artery	N
^15^	30	F	Right forehead FG		R-ICA	N
^16^	33	F	Glabella FG		L-ICA, L-MCA-M1	N
^16^	25	F	Glabella FG		L-ACA, L-MCA-M3	N
^16^	24	F	Periocular FG		L-ACA, R-ICA, R-MCA-M1	N
^16^	19	M	Glabella FG		L-MCA-M1	N
^16^	25	F	Glabella FG		Partial occlusion L ophthalmic artery	N
^17^	25	F	C-section	N	L-MCA-M1	Y
^10^	30	F	Bilateral temporal FG	N	R-ICA-C2, R-ECA	N
^18^	80	M	Revision hip surgery	Y	R-MCA-M1	N
^19^	41	M	Spontaneous		L-MCA	N

Abbreviations: ACA = anterior cerebral artery; ECA = external carotid artery; F = female; FG = fat graft; ICA = internal carotid artery; KR = knee replacement; L = left; LS = liposuction; M = male; MCA = middle cerebral artery; MVR = mitral valve replacement; N = no; R = right; THA = total hip arthroplasty; THR = total hip replacement; Y = yes.

**Table 3.** Treatment technique and device used, timeframe of treatment, initial and final NIHSS and final mRS for each case.

Reference number	Treatment—A or A + S	Device	Symptom onset to recanalization time	Initial NIHSS	Final NIHSS	mRS
^2^	A + S			24^a^	Died	6
^3^	A			24	19^a^	5^a^
^3^	A + S			19^a^		3^a^
^5^	A			15^a^		
^6^	A + S			18	1	
^11^	A + S			4	4	1
^12^	A	Penumbra ACE68 reperfusion catheter, Marksman microcatheter	3 hours	10^a^	4^a^	1^a^
^12^	A	Penumbra ACE68 reperfusion catheter, Marksman microcatheter		5^a^	2^a^	2^a^
^13^	A + S			25	9	2
^14^	Unclear			33^a^	Died	6
^15^	Unclear				Died	6
^16^	A + S	Solitaire		13^a^	7^a^	
^16^	A + S	Solitaire		14^a^	3^a^	
^16^	A + S	Solitaire		15^a^	Died	6
^16^	A + S	Solitaire		22^a^	11^a^	
^16^	A + S	Solitaire		0	0	
^17^	A + S			22		4
^10^	A + S			20	Died	6
^18^	A + S	Solitaire AB		20^a^	12^a^	6
^19^	A		3hr 45 mins	16	8	1^a^

Abbreviations: A = aspiration; S = stent retriever.

a NIHSS/mRS determined from available clinical information.

### Characterizing the CFE cases treated with mechanical embolectomy from the literature

Overall, the review yielded 14 studies with 20 independent cases of CFE treated with mechanical embolectomy. The average age of patients was 44.1±21.6, with a sex distribution of 11/20 (55%) female, 7/20 (35%) male, and 2/20 (10%) unreported. In terms of cause, plastic surgery represented the most common cause, with 10/20 (50%) of fat embolisms being triggered by this, followed by 4/20 (20%) due to orthopaedic surgery, 3/20 (15%) spontaneous embolisms, 2/20 (10%) due to cardiothoracic surgery and 1/20 (5%) due to a caesarean section. Out of 20 cases, 4 (20%) had a confirmed PFO, with 5/20 (25%) having a confirmed absence and 11/20 (55%) not reporting the PFO status of the patient. In terms of location, fat embolisms were found in the following areas: 13/20 (65%) of cases had an occlusion in the MCA, 9/20 (45%) in the ICA, 2/20 (10%) in the ACA, 2/20 (10%) in the ECA, 3/20 (15%) in the basilar artery, 1/20 (5%) in the vertebral artery, and 1/20 (5%) in the ophthalmic artery. Out of 20 cases 5% had fat emboli found in the systemic circulation. The overall mortality was 6/20 (30%).

### Comparing cases treated with aspiration alone or aspiration plus a stent retriever

Out of the cases in the literature, 13/20 (65%) were treated with aspiration plus a stent retriever, 5/20 (25%) were treated with aspiration alone and 2/20 (10%) did not specify the embolectomy method.

The baseline characteristics of the aspiration plus stent retriever cohort and the aspiration only cohort were as follows: The average age of those patients who had a stent retriever was 38.5 ± 21.0 years old, compared to 56.2 ± 19.7 old for those treated with just aspiration (*P* = .0556). There was no statistically significant difference between the gender distributions of the aspiration plus stent retriever vs aspiration only cohort (8/12 vs 1/5 female, *P = .*1312).

In terms of outcomes, 3/13 (23%) patients treated with a stent retriever died compared to 0/5 (0%) patients treated with aspiration alone. The results were not statistically significant possibly due to small sample size (*P = .*5221). Furthermore, the proportion of patients with a final mRS of 0-2 for patients treated with aspiration plus a stent retriever was 25% (2/8, 6 not recorded), compared to 75% (3/4, 1 not recorded) for those treated with aspiration alone, with this difference also not statistically significant (*P = .*1152). Out of those who survived, the average decrease in NIHSS for patients treated with aspiration plus a stent retriever was also not statistically significantly different to those treated with aspiration alone (8.6 ± 6.0 vs 5.5 ± 1.8, *P = .*3771).

## Discussion

Here, we discussed the case study of an elderly lady who presented with a CFE after an elective right total hip replacement. Mechanical embolectomy with aspiration alone was used to treat the condition, and overall, a recovery was seen (*albeit* incomplete). A systematic literature review found 20 cases of CFE treated with mechanical embolectomy, 13 with a stent retriever and 5 with aspiration alone. Overall, although there was a lower mortality rate in patients treated with aspiration alone, this was not statistically significant. Furthermore, there was no significant difference in other outcomes between the 2 cohorts, specifically change in NIHSS and final mRS. This was on the background of no differences in assessed baseline characteristics (ie, age and gender) between the 2 groups. However, various limitations of this review mean these results should be treated tentatively and a difference in outcome could still exist.

Previous literature reviews on CFE have not focussed on treatment with mechanical embolectomy. For example, Ooi et al^3^ reviewed the literature on CFE generally, without a focus on mechanical embolectomy. Other reviews focussed on specific causes of CFE rather than specific treatments: Vetrugno et al^20^ reviewed CFE after traumatic bone fractures specifically and Wang et al^21^ did the same for facial filler injections. Hence, to our knowledge, our study not only provides a new case report for the expanding CFE literature but also represents the first systematic literature review into the use of mechanical embolectomy in CFE.

Our review has several limitations. Firstly, the rare nature of CFE meant only 20 published cases were found. This limits the power of any analysis, meaning statistically significant differences in outcomes between aspiration alone and aspiration plus a stent retriever may have been missed. Furthermore, many values (ie, NIHSS or mRS) were not explicitly stated in papers, meaning they were calculated using descriptive information available from the paper—this could lead to inaccuracies in these values and hence analysis. In addition, comparing embolectomies which were conducted in different centres of course means confounding variables which may have affected outcomes are not controlled for. Furthermore, the patient cohort was heterogeneous with respect to technique, and differences in baseline patient characteristics between cohorts may have contributed to the observed differences in outcomes. Another important limitation of this systematic review is the temporal variability of the technique used in the included cases. The reports span a period from 2009 to 2023, during which time significant advances occurred in thrombectomy device technology. Older cases may reflect practices and devices no longer in current use, such as early-generation aspiration catheters or stent retrievers, which limits the interpretation of technique-based comparisons. The presented comparison must be interpreted with caution. The rarity of CFE inherently restricts the volume of available data, and further case reports or pooled multicentre registries may be necessary to clarify optimal treatment strategies.

Macro-emboli causing CFE can be associated with micro-emboli which have a deleterious effect on cognitive function through triggering an inflammatory process.^22^ Mechanical embolectomy would be unable to remove these microemboli, limiting its ability to reduce neurological damage. The presence of microemboli could explain the lack of recovery of some patients (Tables 1 and 2).

Medically, the use of corticosteroids has been proposed to reduce capillary permeability and hence the effects of CFE.^23^ Additionally, heparin stimulates lipase activity and may help with managing the condition.^24^

Our literature review suggests an overall 30% mortality for patients with CFE managed with mechanical embolectomy. A previous review highlighted a mortality rate of 71% for CFE cases managed without endovascular techniques.^3^ This could suggest that mechanical embolectomy confers a survival benefit compared to non-endovascular techniques. However, due to the low number of cases, the heterogeneity of cases and a lack of randomized controlled trials, it would be premature to make a definitive conclusion at this stage.

CFE leading to large vessel occlusion is rare and our systematic review highlights the clinical contexts and endovascular techniques used in reported cases. We provide a descriptive comparison of aspiration and stent-retriever approaches which suggests aspiration may be superior. However, we emphasize that no firm conclusions regarding procedural superiority can be drawn due to the small number of cases and temporal variability in device technology. Further prospective data are needed to inform evidence-based guidelines.

## Learning points

- Cerebral fat embolism is a rare cause of acute large vessel occlusion and has been reported after orthopaedic surgery, major trauma with open fractures, and plastic surgery.
- Mechanical embolectomy is a feasible treatment option for cerebral fat embolism, and aspiration alone may be effective as a first-line approach.
- Current evidence is limited to case reports and small case series, so the optimal endovascular technique remains uncertain; however, the available data suggest that an aspiration-first strategy is reasonable.

## Supplementary Material

uaag019_Supplementary_Data
